# Royal Albert Orphan Asylum

**Published:** 1889-01-05

**Authors:** Richard Witherby

**Affiliations:** Secretary


### ROYAL ALBERT ORPHAN ASYLUM.

By Richard Witherby, Secretary.

This institution is not valued as it should be. It is not generally known that a widow can get a child of either sex into the Royal Albert Orphan Asylum at Bagshot, without any expense to herself or her friends. That is to say, instead of running into debt for £20 or £30 value of postage stamps and printing, and urgently imploring everybody everywhere to help the most deserving case ever known in history. She describes quietly on a form what her circumstances are, without any blatant particulars of income, and they are placed before the committee. If they consider the case consonant with their rules, they state the particulars for the forthcoming election list—the contributor's initial, such as they think most deserving—send back the papers to the secretary at 62, King William Street, E.C., and they are "boxed" till election day or the day before (to save pressure). There is not, nor can there be, any favouritism. Those in charge of the box only know cases by numbers, and a very clear wish is expressed by advertisement that any governors who would assist in the arduous work of scrutiny would attend to supervise. On the completion of the summing up of the totals the elected are informed thereof by post, and the "nearest five" unsuccessful cases are also informed that they, according to rules, have a second chance There has hardly ever been known a case of canvassing. Should such come before the committee it would be at once removed from the list; but it is remotely likely, as, out of 4,000 contributors and voters, no addresses being given, it would not be likely anyone would trouble to "*tout*" for votes. The in-coming of children is thus briefly described: The out-going after a careful training in education of plain reading, writing, and figures, is highly satisfactory, the sub-Inspector of Government schools (H. E. Dugard, Esq.) testifying highly to the result. The system of half-time, viz., half education, half technical, is invaluable. Hardly a child goes out unarmed with usefulness. Carpenters, tailors, shoemakers, gardeners, farmers, bakers, and band-boys are all sought for—and housemaids. Needlework, etc., and excellent cleanliness and neatness in bed-rooms are a feature among girls over and above their educational teaching. These boys and girls are not necessarily raised above the sphere of life into which they were born, nor above that in which it has pleased God to place them for their future on leaving the care of the Committee of Management. They go out after a careful culture with a desire to get on, and their pride is to write back to those who have trained them to show their success. Nothing pleases them more than to have a run down to the "old quarters," and certainly nothing delights the committee more than to hear of their getting on, or to read their grateful letters of thanks. The fine working of this system of caring for and encouraging the election of children by the non-canvassing system can be readily obtained by calling upon or writing to the Secretary, 62, King William Street, E.C.

